# Supplementary material for: Evidence for continual hybridization rather than hybrid speciation between Ligularia duciformis and L. paradoxa (Asteraceae)
Source: PeerJ. 2017 Oct 11;5:e3884. doi: 10.7717/peerj.3884 (PMC5640982; doi:10.7717/peerj.3884)
Supplement: Table S1 — Leaf samples and voucher specimens were collected from two naturally occurring admixed-growing taxa of L. duciformis, L. paradoxa and their putative hybrids in two hybrid zones (Mt. Maoniu and Heihai Lake, Ninglang County, Yunnan of China) between August 2013 and June 2014. These putative hybrids were morphological intermediates and variant individuals, and the degrees of cracking palmatisect of the leaf blades were different, ranging from lobed to deeply lobed. The suspected backcrossing individuals were found and their morphologies were close to one of the parents. The sympatric species, L. lamarum, was also collected from these two locations to confirm whether it participated in the hybridization. [file peerj-05-3884-s001.docx]

**Table S1** Details of taxon’s sample locations and sample size (n) of two regions

| Locality | Latitude (N)/  Longitude (E) | Altitude (m) | Taxa | Vouchers | Sampling | Labels |
| --- | --- | --- | --- | --- | --- | --- |
| 1. Mt. Maoniu,   Ninglang  (Yunnan) | 100.55˚N  27.81 ˚E | 4040 | *Ligularia duciformis* | X. Gong 22355 (KUN) | 15 | MD1, MD2, MD3, MD4, MD5, MD6, MD7, MD8, MD9, MD10, MD11, MD12, MD13, MD14, MD15 |
|  |  |  | *L. paradoxa* | X. Gong 22356 (KUN) | 20 | MP1, MP2, MP3, MP4, MP5, MP6, MP7, MP8, MP9, MP10, MP11, MP12, MP13, MP14, MP15, MP16, MP17, MP18, MS, MX |
|  |  |  | *L*. × *maoniushanensis* | X. Gong 22357 (KUN), PG130804 (suspected backcrossing individuals) | 9 | MM1, MM2, MM3, MM4, MM5, MM6, MM7, MM8, MM9 |
|  |  |  |  |  |  | ML1, ML2, ML3, ML4, ML5, ML6, ML7 |
|  |  |  | *L*. *lamarum* | PG140601 (KUN) | 7 |  |
| 1. Heihai Hu,   Ninglang  (Yunnan) | 100.53˚N  27.81 ˚E | 4140 | *L. duciformis* | PG130801 (KUN) | 20 | HD1, HD2, HD3, HD4, HD5, HD6, HD7, HD8, HD9, HD10, HD11, HD12, HD13, HD14, HD15, HD16, HD17, HD18, HD19, HD20 |
|  |  |  | *L. paradoxa* | PG130803 (KUN) | 20 | HP1, HP2, HP3, HP4, HP5, HP6, HP7, HP8, HP9, HP10, HP11, HP12, HP13, HP14, HP15, HP16, HP17, HP18, HP19, HP20 |
|  |  |  | *L*. × *maoniushanensis* | PG130807 (KUN) | 10 | HM1, HM2, HM3, HM4, HM5, HM6, HM7, HM8, HM9, HM10 |
|  |  |  | *L*. *lamarum* | -- | 6 | HL4, HL5, HL6, HL7, HL8, HL10 |

“—” indicates loss, voucher of *L*. *lamarum* was not collected as it had the number of less. And these individuals had no flowers or fruits then at the time.

KUN: Herbarium, Kunming Institute of Botany, Chinese Academy of Sciences.
