# Supplementary material for: Evidence for continual hybridization rather than hybrid speciation between Ligularia duciformis and L. paradoxa (Asteraceae)
Source: PeerJ. 2017 Oct 11;5:e3884. doi: 10.7717/peerj.3884 (PMC5640982; doi:10.7717/peerj.3884)
Supplement: Table S2 [file peerj-05-3884-s002.docx]

| accession | No. of cloned sequencing | accession | No. of cloned sequencing | accession | No. of cloned sequencing |
| --- | --- | --- | --- | --- | --- |
| MD1 | 0 | MM2 | 2 | HP2 | 0 |
| MD2 | 0 | MM3 | 4 | HP3 | 2 |
| MD3 | 0 | MM4 | 4 | HP4 | 0 |
| MD4 | 0 | MM5 | 9 | HP5 | 0 |
| MD5 | 2 | MM6 | 3 | HP6 | 0 |
| MD6 | 0 | MM7 | 5 | HP7 | 0 |
| MD7 | 5 | MM8 | 2 | HP8 | 0 |
| MD8 | 2 | MM9 | 2 | HP9 | 0 |
| MD9 | 0 | ML1 | 0 | HP10 | 2 |
| MD10 | 0 | ML2 | 0 | HP11 | 0 |
| MD11 | 2 | ML3 | 0 | HP12 | 0 |
| MD12 | 0 | ML4 | 0 | HP13 | 0 |
| MD13 | 0 | ML5 | 0 | HP14 | 0 |
| MD14 | 2 | ML6 | 0 | HP15 | 0 |
| MD15 | 2 | ML7 | 0 | HP16 | 0 |
| MP1 | 0 | HD1 | 0 | HP17 | 0 |
| MP2 | 0 | HD2 | 0 | HP18 | 0 |
| MP3 | 0 | HD3 | 0 | HP19 | 0 |
| MP4 | 0 | HD4 | 0 | HP20 | 0 |
| MP5 | 2 | HD5 | 2 | HM1 | 11 |
| MP6 | 0 | HD6 | 4 | HM2 | 3 |
| MP7 | 0 | HD7 | 2 | HM3 | 6 |
| MP8 | 0 | HD8 | 0 | HM4 | 6 |
| MP9 | 2 | HD9 | 0 | HM5 | 2 |
| MP10 | 0 | HD10 | 2 | HM6 | 4 |
| MP11 | 0 | HD11 | 0 | HM7 | 3 |
| MP12 | 2 | HD12 | 2 | HM8 | 5 |
| MP13 | 0 | HD13 | 0 | HM9 | 5 |
| MP14 | 0 | HD14 | 3 | HM10 | 7 |
| MP15 | 0 | HD15 | 2 | HL4 | 0 |
| MP16 | 0 | HD16 | 2 | HL5 | 0 |
| MP17 | 0 | HD17 | 0 | HL6 | 0 |
| MP18 | 0 | HD18 | 5 | HL7 | 0 |
| MS | 0 | HD19 | 2 | HL8 | 0 |
| MX | 2 | HD20 | 0 | HL10 | 0 |
| MM1 | 4 | HP1 | 0 |  |  |

**Table S2 The number of clones sequenced for each accession**
