# Supplementary material for: Evidence for continual hybridization rather than hybrid speciation between Ligularia duciformis and L. paradoxa (Asteraceae)
Source: PeerJ. 2017 Oct 11;5:e3884. doi: 10.7717/peerj.3884 (PMC5640982; doi:10.7717/peerj.3884)
Supplement: Table S3 [file peerj-05-3884-s003.docx]

**Table S3. The sites of variation and indels of three cpDNA sequences in related materials**

| Primers/Sites | *psb*A-*trn*H | | |  | *trn*L-*rpl*32 | | | | | | | | | | |  | *trn*Q-5’*rps*16 | | | | |
| --- | --- | --- | --- | --- | --- | --- | --- | --- | --- | --- | --- | --- | --- | --- | --- | --- | --- | --- | --- | --- | --- |
|  | 75  ~  100 | 260 | 300 |  | 16 | 58  ~  121 | 247  ~  248 | 389  ~  394 | 417  ~  427 | 708  ~  712 | 29 | 532 | 563 | 646 | 707 |  | 436 | 437  ~  438 | 366 | 423 | 784 |
| **H1**(MD1~MD15、HD1~HD19) | ① | T | A |  | -- | — | ④ | -- | -- | -- | T | C | T | A | C |  | -- | ⑨ | G | T | A |
| **H1**(HM1、HM3、HM4、HM5、HM7、HM10) | ① | T | A |  | -- | — | ④ | -- | -- | -- | T | C | T | A | C |  | -- | ⑨ | G | T | A |
| **H2**(HD20) | -- | G | G |  | -- | — | ④ | -- | -- | -- | T | C | T | A | C |  | -- | ⑨ | G | T | A |
| **H3**(HL4~HL10) | -- | G | G |  | ② | ③ | -- | -- | ⑥ | -- | C | A | A | A | A |  | -- | -- | T | T | C |
| **H4**(MP1~MP18、MS、MX、HP1~HP20) | -- | G | G |  | -- | ③ | -- | ⑤ | ⑥ | ⑦ | C | C | A | T | A |  | ⑧ | ⑨ | T | G | C |
| **H4**(MM1~MM9、HM2、HM8、HM9) | -- | G | G |  | -- | ③ | -- | ⑤ | ⑥ | ⑦ | C | C | A | T | A |  | ⑧ | ⑨ | T | G | C |
| **H5**(HM5) | ① | T | A |  | -- | ③ | -- | ⑤ | ⑥ | ⑦ | C | C | A | T | A |  | -- | ⑨ | G | T | A |
| **H6**(ML1~ML7) | -- | G | G |  | -- | ③ | -- | -- | ⑥ | -- | C | A | A | A | A |  | -- | -- | T | T | C |

Note: —, delection;

Sequences ①：CTAGTATAGTAAGTATTATCTAGTAT Sequences ②：A

Sequences ③：AAGTTAATTCAATCAAATAAGTTTTTTTTAGTAAAGATTCAAATTGATAAAAAAACTAGTTCAA

Sequences ④：TT Sequences ⑤：ATAATA

Sequences ⑥：ATGTTAAATAC Sequences ⑦：TAATA

Sequences ⑧：G Sequences ⑨：TT
