# Supplementary material for: Evidence for continual hybridization rather than hybrid speciation between Ligularia duciformis and L. paradoxa (Asteraceae)
Source: PeerJ. 2017 Oct 11;5:e3884. doi: 10.7717/peerj.3884 (PMC5640982; doi:10.7717/peerj.3884)
Supplement: Table S4 [file peerj-05-3884-s004.docx]

| K | L(K) | Stdev | L′(K) | [L″(K)] | DeltaK |
| --- | --- | --- | --- | --- | --- |
| 1 | -2787.1 | 0.64 |  |  | 0 |
| 2 | -2414.63 | 0.51 | 372.47 | 292.9 | 574.31373 |
| 3 | -2335.07 | 1.43 | 79.56 | 15.4 | 10.769231 |
| 4 | -2270.95 | 15.33 | 64.12 | 1.3 | 0.084801 |
| 5 | -2208.09 | 1.3 | 62.86 | 49.8 | 38.307692 |
| 6 | -2195.02 | 10.79 | 13.07 | 6.8 | 0.6302132 |

| **Table S4** Analysis of appropriate K value for the SSR data of three *Ligularia*  taxa on the Mt. Maoniu and Heihai Lake sampling sites. |  |
| --- | --- |
