# Supplementary material for: Evidence for continual hybridization rather than hybrid speciation between Ligularia duciformis and L. paradoxa (Asteraceae)
Source: PeerJ. 2017 Oct 11;5:e3884. doi: 10.7717/peerj.3884 (PMC5640982; doi:10.7717/peerj.3884)
Supplement: Table S5 [file peerj-05-3884-s005.docx]

| K | Mean L(K) | Stdev | L′(K) | [L″(K)] | DeltaK |
| --- | --- | --- | --- | --- | --- |
| 1 | -1291.59 | 0.92 |  |  | 0 |
| 2 | -1070.02 | 0.40 | 221.57 | 177.78 | 444.45 |
| 3 | -1026.23 | 0.59 | 43.79 | 26.78 | 45.389831 |
| 4 | -1009.22 | 16.26 | 17.01 | 53.65 | 3.299508 |
| 5 | -1045.86 | 29.70 | -36.64 | 9.37 | 0.3154882 |
| 6 | -1091.87 | 46.67 | -46.01 | 31.12 | 0.6668095 |

**Table S5** Analysis of appropriate K value for the SSR data of three *Ligularia* taxa

on the Mt. Maoniu sampling site.
