# Supplementary material for: Evidence for continual hybridization rather than hybrid speciation between Ligularia duciformis and L. paradoxa (Asteraceae)
Source: PeerJ. 2017 Oct 11;5:e3884. doi: 10.7717/peerj.3884 (PMC5640982; doi:10.7717/peerj.3884)
Supplement: Table S6 [file peerj-05-3884-s006.docx]

| K | Mean L(K) | Stdev | L′(K) | [L″(K)] | DeltaK |
| --- | --- | --- | --- | --- | --- |
| 1 | -1397.00 | 1.60 |  |  | 0 |
| 2 | -1249.24 | 0.70 | 147.8 | 133.9 | 203.22855 |
| 3 | -1235.33 | 3.40 | 13.9 | 40.6 | 11.84248 |
| 4 | -1262.00 | 31.20 | -26.7 | 8.5 | 0.273995 |
| 5 | -1297.20 | 22.20 | -35.2 | 18.8 | 0.845875 |
| 6 | -1351.10 | 24.90 | -53.9 | 34.8 | 1.398659 |

**Table S6** Analysis of appropriate K value for the SSR data of three *Ligularia* taxa

on the Heihai Lake sampling site.
