# Supplementary figures and images for: Evidence for continual hybridization rather than hybrid speciation between Ligularia duciformis and L. paradoxa (Asteraceae)
Source: PeerJ. 2017 Oct 11;5:e3884. doi: 10.7717/peerj.3884 (PMC5640982; doi:10.7717/peerj.3884)

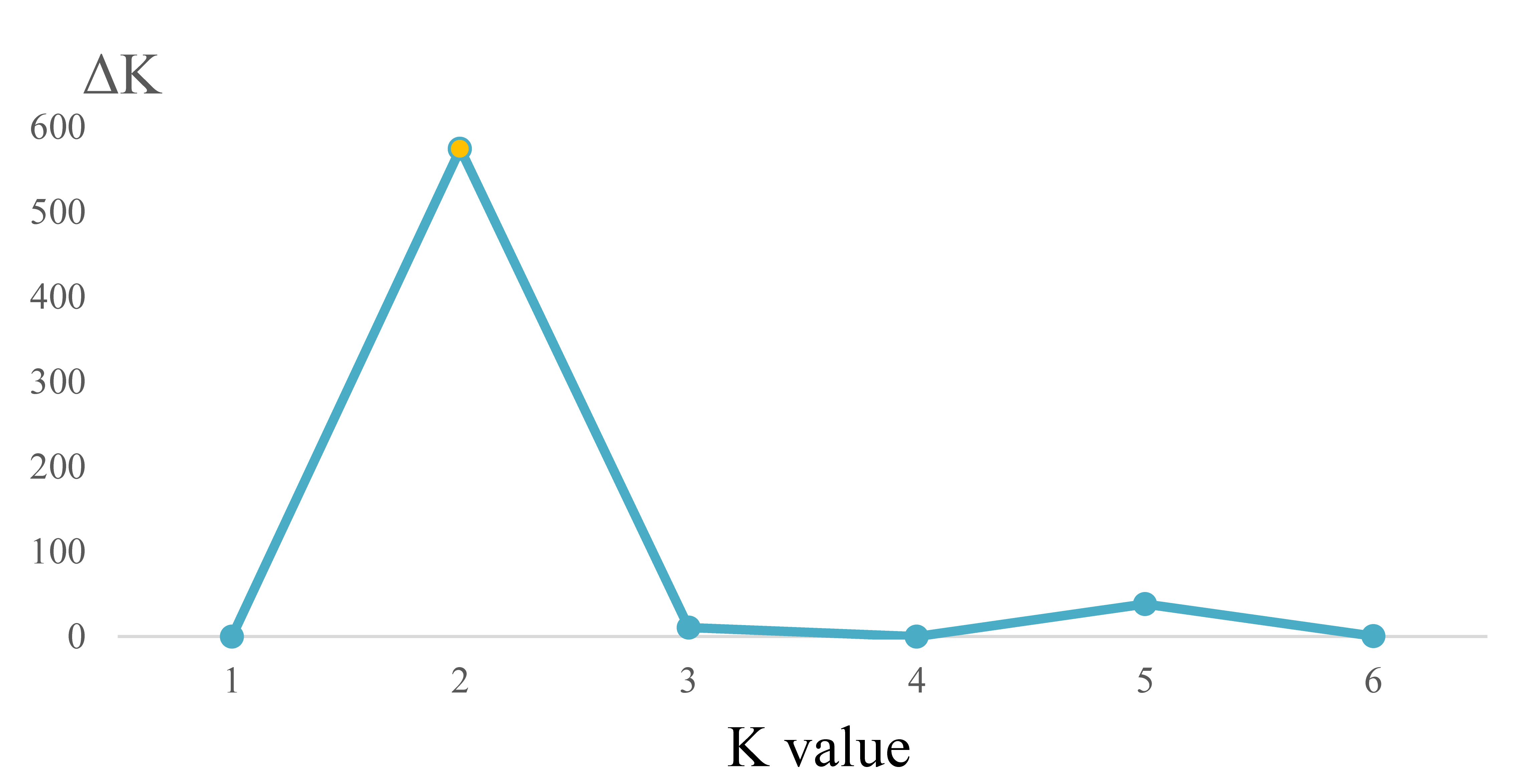

Supplement: Figure S1 [file peerj-05-3884-s007.png]

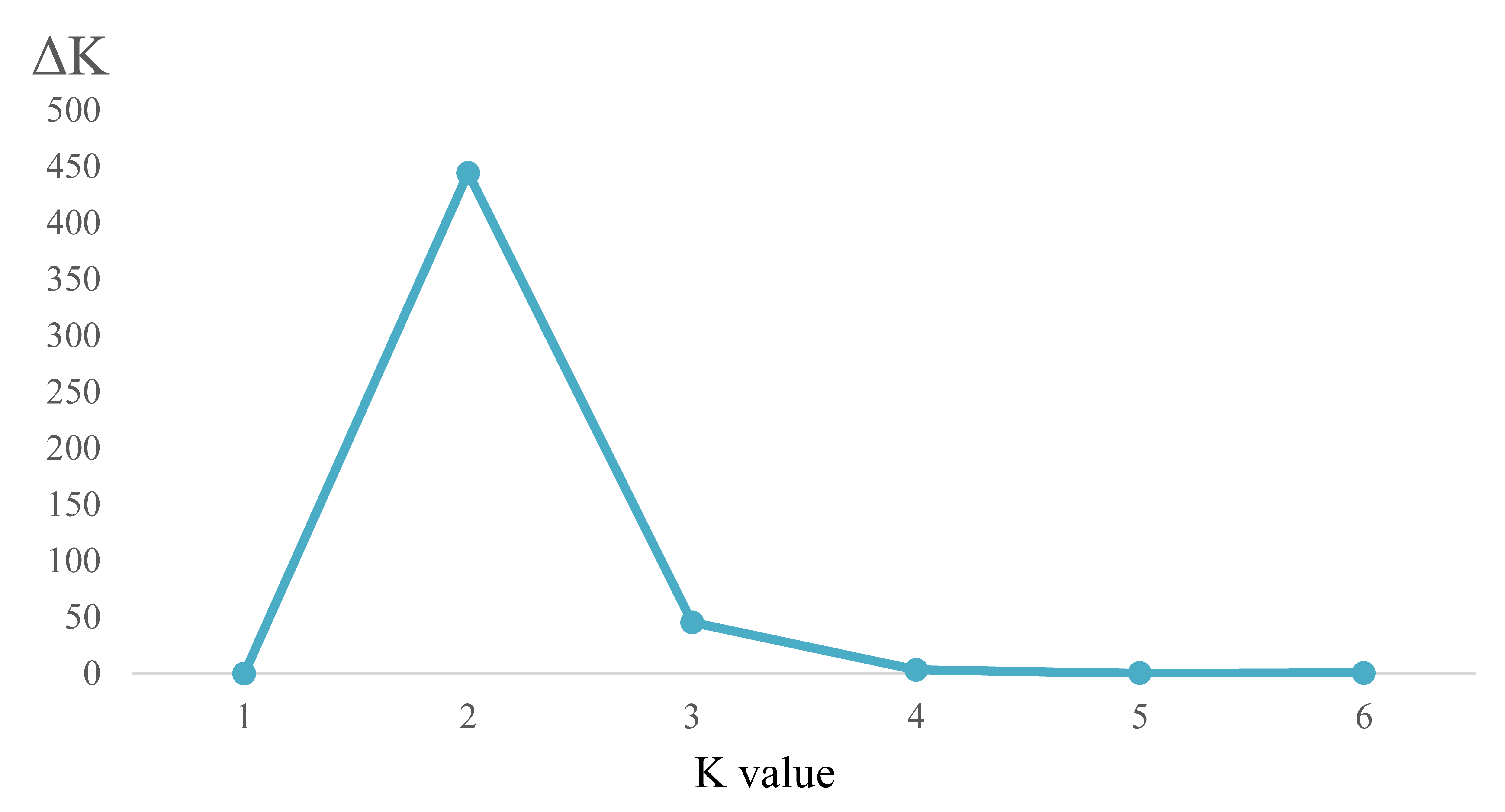

Supplement: Figure S2 [file peerj-05-3884-s008.png]

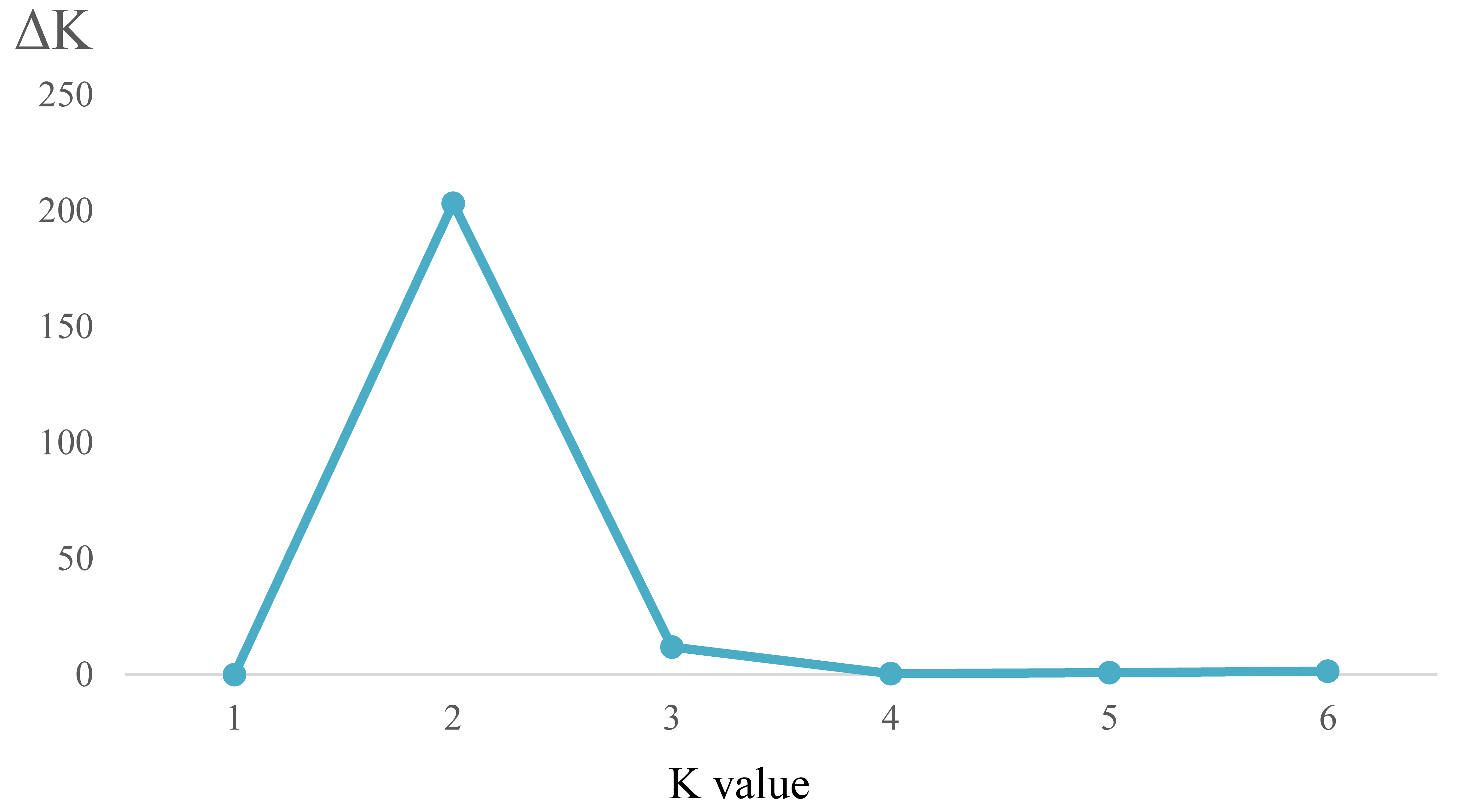

Supplement: Figure S3 [file peerj-05-3884-s009.png]

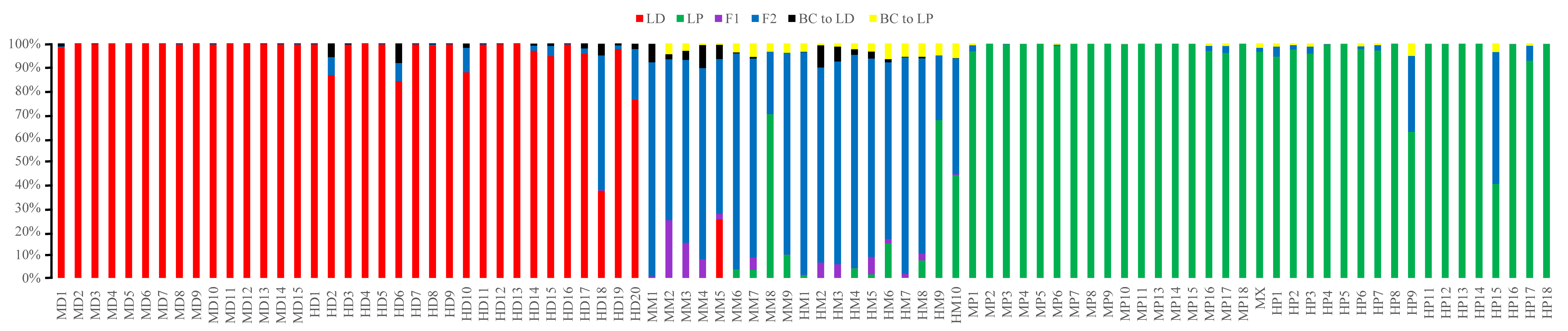

Supplement: Figure S4 — NewHybrids analysis was implemented for 89 individuals from the two sampling sites showing slightly different results of join. All the samples are represented as a vertical bar partitioned into segments whose length is proportional to the likelihood of belonging to a certain class. MD and HD, MM and HM, MP and HP represent morphologically identified L. duciformis (LD), L. ×maoniushanensis and L. paradoxa (LP), respectively. M and H represent the two hybrid zones, Mt. Maoniu and Heihai Lake, respectively. [file peerj-05-3884-s010.png]
